# *Technical note*:Beyond Good Laboratory Practice

**DOI:** 10.1155/S1463924692000361

**Published:** 1992

**Authors:** Dick Le Vitt

**Affiliations:** Analytical Group Quality Manager Hewlett-Packard Co. USA


Journal of Automatic Chemistry, Vol. 14, No. 5 (September–October 1992), pp. 189–191

*Technical note*

### Beyond Good Laboratory Practice

**Dick Le Vitt**
*Analytical Group Quality Manager, Hewlett-Packard Co.*

### Introduction

'Good Laboratory Practice' (GLP) refers to a set of government regulations for non-clinical testing. The phrase was first used in a regulatory context in New Zealand, where in 1972 the Testing Laboratory Act specified conditions for planning, performing and recording studies so that results are reliable. Later, the US Food and Drug Administration (FDA), followed by the Environmental Protection Agency (EPA), developed GLP regulations covering chemical safety and efficacy testing. The Organization for Economic Cooperation and Development (OECD) and then the European Community (EC) required member states to adopt GLPs that are closely related to those of the FDA and EPA. Many nations, including the UK, Germany, France, Italy and Japan, are in accord with the OECD/EC guidelines.

The primary motive behind these regulations is public safety. Government agencies around the world depend on industrial laboratories for test results on medicines, pesticides, and other products that may have harmful effects on people or the environment. Unlike pure research, the work of these laboratories is not independently duplicated; agencies must trust in the initial accuracy of studies that are funded by industrial sponsors.

This trust was violated in the 1970s. Evidence of fraud and error prompted the FDA, EPA and OECD to control testing practice and improve the quality and integrity of test data. A series of GLPs were created that have been refined and extended through the years. The experience of the agencies and participating laboratories has resulted in a set of truly good practices that have value for any testing enterprise. GLPs are particularly useful when critical decisions depend on the accuracy and trustworthiness of results.

Despite the success of GLPs, however, they do not completely cover the notion of 'good' in a modern laboratory. This article highlights some limitations of the original GLP regulations. The new EPA Good Automated Laboratory Practices are described, and it is explained how these, combined with state-of-the-art quality techniques, can carry laboratory managers *beyond* good laboratory practice.

### Scope of GLP

In regulatory language, good laboratory practice is a management system to ensure that experimental studies produce authentic results. These results must be independently verifiable by a detailed audit of experiment plans, observational methods and raw data. To be in compliance with GLP regulations, a study must:

(1) Follow a documented experiment plan or protocol.
(2) Be conducted by appropriately qualified people.
(3) Conform to documented Standard Operating Procedures (SOPs).
(4) Be conducted in appropriate facilities.
(5) Use carefully calibrated and maintained equipment.
(6) Have original observations – raw data – archived and available for inspection.

When verifying compliance to GLPs, agencies rely heavily on two levels of inspection. The first level is internal to the regulated laboratory. An independent Quality Assurance Unit (QAU) must review each study for compliance with GLP requirements. All reviews must be reported to management and documented along with records of discrepancies found and corrective action taken. The second level is an agency inspection. An agency may perform a detailed inspection of any laboratory over which it has regulatory jurisdiction. Included in the audit is a review of the internal QAU. In addition, an agency may audit an experiment in depth from raw data through final report.

These requirements define 'goodness' from a regulatory point of view, but, as broad as they seem, they are incomplete. The regulations do not address all aspects of quality assurance in the laboratory.

The limitations include:

(*a*) No guidance on the quality of the science. Though GLPs require a documented protocol, they have nothing to say about the effectiveness of the protocol, the power of the statistical methods used or the efficiency of the experiment design. Under GLP regulations, it is possible to conduct precisely a set of useless tests.

(*b*) Little attention to process improvement. GLPs require that all deviations from experimental protocols or SOPs be documented along with the rationale for change. Instrument problems, human errors and retests must be recorded as well. However the GLPs treat these as isolated events to be logged for audit purposes, not as measures of process performance. A process management focus would encourage statistical process control methods. Process thinking leads to root cause analysis of problems and systematic elimination of error in future experiment runs.

(*c*) An emphasis on inspections to guarantee test quality. Like any other complex human activity, inspections

0142–0453/92 $3.00 © 1992 Taylor & Francis Ltd.

are subject to error. It is unrealistic to assume that QAUs or agencies are capable of detecting all defects in the large-scale studies done by today's chemical and pharmaceutical industries. QAUs can typically do no more than sample the work of their labs. The FDA and EPA, though they aim for consistency, cannot completely eliminate the effects of individual differences among their inspectors. The outcome of an audit is partly a function of the people who do the inspecting. Quality emerges when people strive continually to improve their tools and processes.

### The process approach to quality

During the 1970s and early 1980s when the GLPs were being formulated, industrial firms in the USA and Europe used a policy of quality management grounded in audits and inspections by third party organizations, the QA or QC units, who did not report to production. This approach became widespread in the years following the Second World War and was adopted by many manufacturers, including Hewlett-Packard.

In the meantime, another approach to quality gained strength among the Japanese. This quality management strategy originated with Walter Shewhart, a US statistician, and came to post-War Japan through the teachings of Deming and Juran. In the following decades, the Japanese demonstrated the superiority of statistical process control. This resulted in the belated recognition of Deming and Juran in the West and led to a revolution in our thinking about quality. Unfortunately, this revolution came too late to influence the authors of GLP regulations.

Under the process approach, work is broken down into stages, each with suppliers (upstream process steps) and customers (downstream process steps). Errors are treated as data, not as cause for blame. Suppliers and customers become partners working together for better results. This quality strategy can yield dividends that accumulate like compound interest. The strategy is so powerful that it is often called 'Total Quality Control' or 'TQC' by its advocates.

In the TQC method, the workers themselves are the primary inspectors. Workers are encouraged to take ownership for quality. They track incoming quality levels, check their own results, and statistically analyse and correct the causes of process problems. In this way, the intrinsic error rate of the process is steadily driven down. As error rates are reduced, the need for independent inspectors is diminished.

This it not to suggest that independent audits should be eliminated. In regulated laboratories, the QAUs and agencies will always be important safety nets for public protection. However, even in regulated industries, TQC is a better strategy than quality assurance through inspections alone. Inspections add cost to any operation. TQC saves cost by eliminating the sources of error.

Of course, process management is not the only factor in Japan's extraordinary success. However, it is an important element in the productivity and quality that nation has achieved. Hewlett-Packard's (HP's) experience verifies this. In the early 1980s YHP, HP's Japanese subsidiary, won the Deming Prize. This achievement inspired other organizations in the company to examine their approach to quality. What followed was a transformation. Hardware failure rates were reduced by a factor of 10 in a decade. Productivity increased: costs arising from waste and rework were reduced. Independent inspections were also eliminated from the majority of HP's operations.

With the advent of Europe 1992, many laboratory managers have become interested in ISO 9000 as a framework for quality management. ISO 9000 is a set of standards that define requirements for a quality system. Although the EC has not universally endorsed ISO 9000, it has adopted the standards as European Norms, and has issued directives affecting a few industries. Customer interest in these standards caused a number of companies, including Hewlett-Packard, to pursue ISO 9000 certification.

ISO 9000 and GLPs are very much alike in their approach to quality. ISO 9000 is generic, to suit virtually any industry, while the GLPs are far more specific. Neither gives attention to statistical process management. Therefore adoption of ISO 9000 in a laboratory will not naturally lead to the changes signalled by Deming and Juran.

The challenge for laboratories is to work within the requirements of GLP to build processes that are both efficient and mistake-proof. People being error prone as they are, automation is important to this task.

### The role of automation

Automated samplers, smart instruments and computer systems are generally thought of as productivity tools. Productivity is a major benefit of these devices. It is less widely recognized that these modern labour-saving technologies are also quality tools. The sampler that injects precisely the same amount time after time minimizes variation in the process and reduces the chance of manual error. An instrument that remembers methods, logs data and diagnoses its own health also reduces error. And the computer system that reads each bar code, tracks each sample history, interrogates each instrument and prepares the report prevents many mistakes that can occur in a manual operation.

The potential that automation brings for laboratory quality improvement has only just begun to be exploited. Many of the repetitive tasks and data logging functions have been taken into the instruments and computer systems. However, these technologies are still not being used to manage the processes using Total Quality Control.

Process management requries a lot of data. The natural tool for collecting and analysing this data is the computer in a unified laboratory. In an ideal laboratory, a Laboratory Information Management System (LIMS)

would track all errors and present the results using control charts, Pareto charts and the other tools of TQC. The database would permit workers to do multilevel queries and rapid diagnosis of roots causes. And SOPs would be on-line and 'instrumented' with process metrics so that a diagnosis could quickly result in changes to standard procedure.

The possibilities are limitless, more than enough to keep LIMS developers, VARs and system administrators busy for some time. However, another regulatory guideline has come along which will actually ease the task of using lab systems for process management. This guideline is GALP, or Good Automated Laboratory Practice.

### Good automated laboratory practice (GALP)

For some years, regulators have realized that the original GLPs do not adequately describe the management of computer systems. To address the issue, the US Drug Information Association published *Computerized Data Systems for Nonclinical Safety Assessment* in 1988. Another attempt was made to define system requirements by the UK Departments of Health in 1989. In 1990, the EPA researched data management practices and their suitability to GLP. This work led to the GALP recommendations which are presently in draft form.

The EPA GALP standard applies the data quality assurance principles of GLP to computer systems. GALP does not replace the GLP, but clarifies and standardizes the interpretation of GLP for computer-resident data. The GALP standard is based on six principles best described by the EPA's implementation guide:

(1) *Data*: The system must provide a method of assuring the integrity of all entered data.

(2) *Formulae*: The formulae and decision algorithms employed by the system must be accurate and appropriate.

(3) *Audit*: An audit trail that tracks data entry and modification to the responsible individual is a critical element in the control process.

(4) *Change*: A consistent and appropriate change control procedure capable tracking the system operation and application software is a critical element in the control process.

(5) *Standard Operating Procedures* (SOPs): Control of even the most carefully designed and implemented system will be thwarted if appropriate user procedures are not followed.

(6) *Disaster*: Consistent control of a system requires the development of alternative plans for system failure, disaster recovery and unauthorized access.

These are strong principles and their importance to LIMS users and developers should be clear. Also clear is the bonus for process management that arises from the demand for data integrity. Data integrity requires audit trails that give insight into errors, retests and other causes for change. All that has gone wrong is on record. When systematically examined, the data kept for GALP compliance becomes a powerful mechanism for improving the intrinsic performance of laboratory processes.

### Conclusion

GALP imposes special requirements on system users and operators to guarantee the integrity of data. The requirements for data entry, audit trails, change control and SOPs mean that histories must be kept of all transactions. These histories must include all errors, rework, retests and the like. This information is a gold mine for process management.

The data that must be preserved under GALP includes the very information needed to track and diagnose process performance. A system managed to GALP will contain records of all operations that change data, together with the reasons for change. This offers an unparalleled opportunity for developers and users to apply the techniques of Total Quality Control toward laboratory management.

Once the initial investment in equipment and training is made for GALP, the added cost for process data is small and the returns could be enormous.

